# Innovating the outreach of comprehensive cancer centers

**DOI:** 10.1002/1878-0261.12451

**Published:** 2019-02-02

**Authors:** Christian H. Brandts

**Affiliations:** ^1^ Department of Medicine Hematology/Oncology University Hospital Frankfurt University Cancer Center Frankfurt (UCT) Germany

**Keywords:** cancer, comprehensive cancer centers, quality assurance

## Abstract

In many countries, a majority of cancer patients are not treated at comprehensive cancer centers. Even for those that are, parts of the treatment or follow‐up may be carried out in local community hospitals or in private practices. How to assure quality in cancer care and create innovation? How to integrate decentralized versus centralized patient care, education, and cancer research? Outlined here is a 360° view of outreach to include all stakeholders—most importantly patients and their families, patient advocacy groups, healthcare providers, health insurers, and policymakers.

**Abbreviations**
CCC: comprehensive cancer center
EGFR: epidermal growth factor receptor
SOP: standard operating procedure

## 1 Challenges

Since the 1970, numerous comprehensive cancer centers (CCCs) have been founded and developed in the United States, followed by similar developments in Europe and throughout the world in the past two decades. At many university hospitals, CCCs evolved as matrix cancer centers and have developed into powerful and efficient institutions to integrate all departments and institutes responsible for patient care, education, and cancer research. CCCs aim to involve all professional groups, from clinicians, scientists, nurses, administrative staff to students—as well as patients, self‐help and advocacy groups. During their evolution, CCCs had to overcome a decentralized, fragmented organization of cancer medicine at each university hospital to transform into a coordinated CCC with balanced centralized and decentralized functionalities. This has led to significant improvements in the quality of multidisciplinary patient care, a stronger integration of translational cancer research approaches, and increases in clinical trial activity as well as interdisciplinary education and training programs. While these are very positive developments, it has been particularly challenging for CCCs to grow in order to provide access for a majority of cancer patients and their families.

There are numerous reasons why a majority of cancer patients are not treated at CCCs. While these are likely to significantly differ from country to country, two reasons are nevertheless commonly identified. First, the total number of CCCs is still too small in most (if not all) countries to cover the majority of the patient population. Particularly in rural areas, access to specialized care of a CCC is often very limited. Secondly, many CCCs are not high‐volume centers because even in the vicinity of a CCC (i.e., within a 1‐h driving distance), the majority of cancer patients are not treated at the CCC itself or only partially for specialized treatment, such as specific surgery or radiation therapy. Instead, a heterogeneous group of competing market players (i.e., private hospitals, large municipal and smaller community hospitals as well as specialists in private practice) are diagnosing and treating the majority of cancer patients in most countries. These competitors often have diverging goals from CCCs, and these developments are largely driven by economic market forces.

As a consequence, spreading innovation to patients can be slowed down and limited, particularly in an era where modern diagnostics (including novel imaging and genomic profiling) are increasingly paired with stratified ‘personalized’ treatment. For example, 10 years after approval of epidermal growth factor receptor (EGFR) inhibitors, EGFR mutation testing in patients with advanced non‐small‐cell lung cancer is still below 75% in Germany. As clinical trials are more and more designed for small subgroups of genetically defined patient populations, they are impossible to perform without a sufficient patient base. Furthermore, as many countries are facing demographic challenges with an aging population and an increase in the incidence of cancer, the hurdles could increase in the decades to come unless new models of cooperation are adopted.

In countries with very centralized cancer care (such as the Netherlands), these challenges may have less impact. However, in countries with very decentralized patient care (such as Germany) the limited number of CCCs and their limited market share have clearly resulted in the following:

- A fragmented multidisciplinary cancer patient care in prevention, early detection, diagnostics, treatment, and follow‐up, with many stakeholders that have overlapping as well as divergent goals.
- A heterogeneity of education and training for doctors, nurses, and other healthcare professionals.
- Limitations in access to innovations and clinical trials.
- Silos of data that are not integrated.

From the viewpoint of CCCs, this significantly limits its impact on quality assurance and spread of innovation. In countries with significant health disparities and large underserved populations, this can have dramatic consequences at a population level, in particular when additional psychological barrieres exist (Fayanju *et al.,* 2014). In a recent report, The Ohio State University CCC surveyed residents in its catchment area to understand the health of this population to tailor outreach and research strategies. Large variations were found in cancer attitudes, smoking and diet, as well as adherence to guidelines for screening, thus requiring new approaches to focus on vulnerable populations (Paskett *et al.,* 2018). Patient advocacy groups, health insurers, and policymakers are increasingly aware of these issues, but are struggling to find answers to these problems.

A novel internal organization within CCCs and increasing network approaches are required to address these challenges.

## 2 Innovative organization to ‘regionalize’ CCCs

The challenges outlined above are not new, and the regional context affects every CCC in a particular way. Therefore, no single organizational solution will fit all. As CCCs have learnt how to balance centralized and decentralized operations within their own institutions, many have built on this experience to develop an organization that does not end at their front door. Similarly, the patient's pathway does not start at the entrance of the CCC, but rather has to be viewed as starting within their local community, with their local general practitioner initiating first diagnostic (and potentially therapeutic) steps.

Overall, an organizational pattern has emerged whereby ‘regionalizing’ many existing features of a CCC can lead to the inclusion of outside partners to improve interaction and work with regional, national, and even international partners. The required organizational changes of the CCC to incorporate the outreach are exemplified in the following sections. Usually, an outreach coordinator or outreach office is required to oversee and monitor the different aspects and layers of outreach activities. Key metrics for each of the features outlined below can be helpful to quantify and monitor outreach developments over time.

### 2.1 Regionalizing multidisciplinary patient care

A traditional and effective way to bridge CCCs and partner institutions in patient care has been to employ doctors and nurses in part‐time at both institutions, thereby fostering exchange. As this is not always possible, additional instruments have been developed in past years.

Weekly tumor boards are the widely accepted instrument for multidisciplinary decision‐making. In municipal or community hospitals, these are often supported by specialists from the CCC that physically attend or are connected by video‐conferencing. In a complementary approach, at least in Germany, patients are increasingly presented to tumor boards at the CCC by external treating physicians (Brandts, 2017). Because physical attendance is not always possible, virtual attendance by video‐conferencing of doctors from surrounding hospitals or practices has been effective to increase the number of patients discussed in a multidisciplinary setting throughout the region (Stevenson *et al.,* 2013). Video‐conferencing is of particular importance for CCCs in rural settings by eliminating travel and shortening evaluation time before treatment (Stevenson *et al.,* 2013).

Standard operating procedures (SOPs) based on international guidelines have been tailored by most CCCs to harmonize the diagnostic and therapeutic procedures for the most frequent cancer entities and guide doctors through them. Making these available to the regional partners has been the first step for many CCCs. However, making these SOPs obligatory for standard care and measuring their adherence have remained difficult.

Co‐certifications of partner sites and peer reviews (with staff from both CCC and partners) can be useful to enforce an obligation to meet equal quality standards at the CCC as well as the partner site. Quality managers from the CCCs can support the quality management of partner institutions in the region and thereby harmonize quality standards. Furthermore, a CCC can strengthen partnerships by the offer of special outpatient services that are beyond the capabilities of their regional partners, such as specialized outpatient clinics for patients with rare cancer entities, second opinions, long‐term survivors, or inclusion in clinical trials.

New diagnostic methods, such as novel imaging and genomic profiling technologies, have to be tested, validated, and integrated into routine patient care. Making these readily available to outreach partners and their patients has been an effective way to strengthen collaboration and move innovations from the CCC to its outreach, as these technologies are usually not available at these sites. An encouraging example is the National Network for Genomic Medicine in Germany, which is offering molecular diagnostics at German CCCs for all patients with advanced lung cancer (www.nNGM.de) and has the potential to serve as a blueprint for diagnostics‐based outreach in Germany. This does, however, require significant investments at the CCCs, which may limit future developments.

Clinical cancer registries are the instrument of choice to evaluate process quality, structural quality, and—most importantly—outcome quality on a regional, national, and international level. Throughout the world, these have very different levels of development. Results from several European countries have clearly demonstrated the added value of measuring quality of patient care in an unparalleled depth. For example, the real‐world data of treatment and outcome of over 50 000 stages I–III rectal cancer patients in eight European countries receiving multimodal treatment were compared (Breugom *et al.,* 2018). Availability and careful analysis of such data within the catchment area of a CCC should allow a detailed view on the regional treatment reality and outcome to devise measures to improve quality of prevention, early diagnosis, and cancer care.

Finally, IT solutions are clearly required to support interaction by transmitting histology, tumor board recommendations, imaging, laboratory results, and correspondence in a bidirectional manner from one hospital information system to the other and to private practices. Effective IT solutions for data exchange with the large number of referring general practitioners are often particularly challenging, as they will only share few patients at a time.

### 2.2 Inclusive education and training programs in multidisciplinary care and research

Doctors, nurses, and other healthcare professionals required for patient care as well as translational and clinical cancer research need continued education and training beyond medical school or nursing school. Continued education programs within CCCs on topics such as multidisciplinary care for different cancer entities or integration of innovative diagnostics and treatment into daily routine are frequently offered to all health professionals at partnering institutions. Training can occur in both directions. For example, in Germany many smaller surgical procedures are performed far more frequently in community hospitals than at CCCs—opening training opportunities for young surgeons from CCCs to receive training at these hospitals. Personnel exchange for training purposes can thereby create opportunities to the mutual benefit. Similarly, extensive information seminars of high quality are offered by CCCs for patients, their families, and self‐help and patient advocacy groups, irrespective of where patients are treated. This should also include high quality websites and a social media presence to help achieve the goals of public education of a broad audience (Huerta *et al.,* 2017). While there are certainly numerous ways how to address these issues, national and regional characteristics will have to be considered for optimal results and will require continued support in order to maintain and develop in the future.

### 2.3 Regionalizing translational and clinical research

Basic and translational cancer research occurs almost exclusively at academic centers such as CCCs. Nevertheless, regional partners can be involved in the procurement of biospecimens (regional biobanking), collection of clinical data in registries to gather real‐world outcome evidence and support for diagnostic and biomarker‐driven research projects. The most important aspect is the clinical trial arena. Clinical trials are increasingly designed for small, genetically defined subgroups of patients that require a large patient base to screen, identify, refer, recruit, and treat in clinical trials. For example, over 6000 adult and pediatric cancer patients were screened to identify and treat 55 patients with NTRK fusion‐positive cancers with larotrectinib (Drilon *et al.,* 2018). This trial demonstrated an extraordinary overall response rate of 75% and led to the approval of this drug, which would not have been possible without effective clinical trial networks. Also, the access of this drug will continue to require large diagnostic networks in order to provide the drug to this specific subgroup of patients. Overall, patient recruitment in clinical trials at CCCs increasingly relies on effective outreach networks. Several models of collaboration are being used, including patient referral to the CCC, satellite centers at partner institutions, and integrated models (with CCC plus one or more partner site acting as one integrated trial center).

Finally, medical informatics approaches are needed to effectively integrate data from various sources at the CCC with data at separate institutions and legal entities, in accordance with data protection laws, to make these data available for a more global analysis of real‐world evidence. Several US and European initiatives are underway to develop the appropriate tools to break up data silos, merge data, analyze it to answer clinically meaningful questions, and fill the existing gap between clinical trials and assessment of effectiveness (Celis and Pavalkis, 2017).

## 3 Building and maintaining networks of collaboration

**Figure 1:**
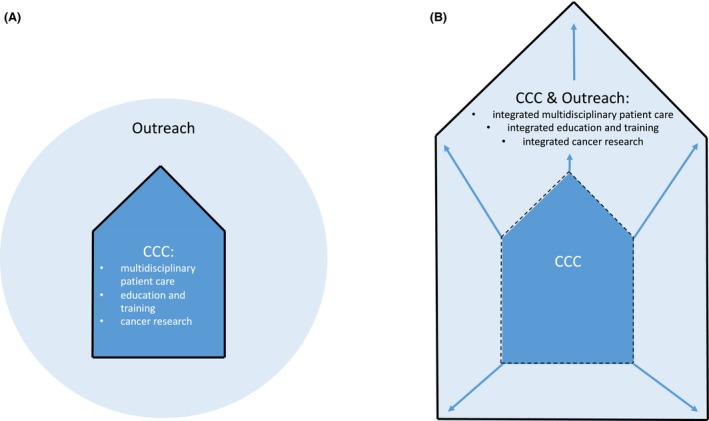
Simplified schematic models. (A) ‘Stand‐alone CCC’ with clear separation of the CCC from the outreach. (B) ‘Matrix CCC’ integrating outreach partners.

Once organizational prerequisites are in place at the CCCs as outlined above, CCCs can transition from a ‘stand‐alone’ CCC to a ‘matrix CCC’ integrating outreach partners. The schematic diagram (Fig. 1) is an oversimplified organizational depiction intended to portray the two possible approaches to view the outreach: High‐volume CCCs will tend to a model as shown in Fig. 1A, while a majority of CCC will need to develop different layers of networks to branch out their different functionalities to the region (Fig. 1B). In the latter ‘inclusive’ model, boundaries of the CCC become more permissive with the intention to bring the inside of the CCC to the outreach, and regional partners into the CCC.

Every country and health system has its own specifications. For instance, many CCCs in the United States have heavily invested to buy or build up their own satellite centers in the larger region in order to provide the full portfolio of primary, secondary, and tertiary care to all cancer patients. Most matrix CCCs with more limited resources face the challenge to develop, maintain, and monitor the large variety of activities with all partners, small and large. This may be viewed as too time‐consuming, but—unless significant resources for investments are available—is without real alternative.

In multidisciplinary care, this can lead to agreements of complementarity, where certain specialties are uniquely offered at the CCC (e.g., bone marrow transplantation or complex clinical trials), while some procedures may be predominantly performed at a partner site (e.g., follow‐up and lower complexity clinical trials). Equally, for education and training purposes as well as for successful clinical trial networks, win‐win situations have to be found with every single partner at regular intervals. In any way, the integrative model (Fig. 1B) requires much tighter collaboration, accountability, and responsibility compared to a loose outreach network with intended collaborations (Fig. 1A). As a consequence, this requires obligatory contractual agreements and monitoring of key metrics in a timely fashion to allow transparency for all partners involved. A prerequisite for any fruitful collaboration remains direct and effective communication skills on all sides with the aim to build trust over time. In the long run, a shared view of maximal benefit for patients and a functioning healthcare system paired with a willingness to collaborate to reach this goal are mandatory requirements.

## 4 Outlook

In order to be successful, innovating outreach for CCCs requires significant resources. Needless to say that the maintenance requires continuous work, as organizations change, people in charge change, their goals change, leading to a constant struggle to find innovative ways to interact. Furthermore, the increasing economic pressures in the healthcare market often lead to opposing effects. Patients and their families as well as self‐help and patient advocacy groups have to be informed about these issues. Health insurers and policymakers, which in principle are very supportive of networks for quality assurance, need to modify regulations in order to support frameworks of collaboration and provide the necessary funding that allows these to thrive. This will, in the long run, support regional, national, and international outreach and collaboration for the better of patient care.

## Conflict of interest

The author declares no conflict of interest.
